# Effects of Physical Exercise on Mobile Phone Addiction in College Students: The Chain Mediation Effect of Psychological Resilience and Perceived Stress

**DOI:** 10.3390/ijerph192315679

**Published:** 2022-11-25

**Authors:** Zitong Zhao, Shuai Zhao, Qi Wang, Yiran Zhang, Chunchun Chen

**Affiliations:** 1 Business School of Sport, Beijing Sport University, Beijing 100084, China; 2 College of Industry and Commerce, Shandong Management University, Jinan 250357, China; 3 School of Management, Beijing Union University, Beijing 100101, China

**Keywords:** physical exercise, psychological resilience, perceived stress, mobile phone addiction

## Abstract

Smartphones have become an integral part of people’s daily lives. While bringing convenience, mobile phone addiction caused by overuse of smart phones has become a common phenomenon among college students. The current study aimed to examine the serial mediating role of psychological resilience and perceived stress between physical exercise and mobile phone addiction of college students. Using the PARS-3 scale, CD-RISC-10 scale, PSS-10 scale, and MPA scale, 257 college students were investigated and Structural Equation Model (SEM) was conducted. The results show that: (1) Physical exercise has no significant direct impact on mobile phone addiction. (2) Psychological resilience has a significant mediating effect between physical exercise and mobile phone addiction. But perceived stress does not. (3) Psychological resilience and perceived stress play a chain mediation role. Physical exercise can enhance psychological resilience firstly, thus relieving perceived stress and eventually mitigating mobile phone addiction.

## 1. Introduction

With the rapid development of mobile information technology, smartphones integrating multiple functions have become indispensable in people’s daily lives. They have penetrated various fields, such as people’s social interactions, entertainment, office work, shopping, and wealth management [1]. According to China Internet Center statistics, the number of Chinese netizens has reached 1.032 billion as of December 2021 [2]. Smartphones may harm people’s physical and mental health despite the convenience they bring to people [3,4], resulting in conditions such as myopia, obesity, insomnia, depression, and anxiety [5,6,7,8]. Mobile phone addiction is the most direct negative impact of smartphones [9], and has become a global problem. Data show that mobile phone addiction is more common among young people, especially college students, where the detection rate is as high as 21.4–27.4% [10,11], making it a widespread phenomenon. The COVID-19 pandemic has exacerbated students’ mobile phone addictions due to the need for online learning and social networking, making “the phone is never separated from the user” a daily habit of college students [12], negatively affecting their lives [13]. Consequently, the issue of college students’ mobile phone addiction warrants our urgent attention and active exploration of mitigating strategies.

Mobile phone addiction has been defined as the excessive use of smartphones and the loss of control over the behavior, characterized by cognitive salience, mood changes, and relapses [14,15,16]. It resembles gambling and video game addictions, with strong psychological and behavioral effects. Addicts with uncontrolled smartphone use often experience decreased life satisfaction [17], depersonalization [18], heightened negative emotions [10], and decreased sleep quality, etc. [19,20]. College mobile phone addicts are especially prone to procrastination, affecting their studies [17]. Hence, the academic community has extensively researched the antecedent variables of mobile phone addiction behavior.

Academic research has focused on individual internal psychological factors such as perceived stress, social anxiety, alexithymia [21,22,23,24,25,26,27], etc. Nevertheless, few studies have focused on how physical exercise may affect college students’ mobile phone addiction behavior, and research on the interactive mechanism between the two is inadequate. Thus, this study takes Chinese college students as the subjects to construct a chain mediation model, introduces psychological resilience and perceived stress into the model, and explores the role they play in physical exercise and mobile phone addiction, providing some references for future studies of college students’ mobile phone addiction.

## 2. Literature Review and Research Hypotheses

### 2.1. Physical Exercise and Mobile Phone Addiction

Physical exercise is physical activity with a certain intensity, frequency, and duration, aiming at enhancing one’s physical health, with body movements as the content and means [28]. In addition to promoting healthy physical development, the impact of physical exercise on mental health cannot be ignored [29]. Numerous studies have shown that appropriate physical activity can reduce people’s psychophysiological responses related to state and trait anxiety, thereby reducing their stress, improving their sense of happiness, and preventing depression in individuals [30,31,32,33].

The uses and gratifications theory (UGT) posits that individuals use new media to satisfy their various needs, which is widely adopted in media communication research [34,35]. According to the theory, the smartphone is a “ritualized” medium used to satisfy people’s needs for passing the time, relaxation, and entertainment, and it is prone to habit-forming [36]. An individual’s psychological needs, such as socializing, entertainment, etc., are satisfied to varying degrees with smartphone use. This sense of satisfaction prompts the individual to depend on network devices such as mobile phones [37], resulting in mobile phone addiction [38,39]. The recreational, emotional-health nurturing, and interpersonal functions of physical exercise [40] can also satisfy the psychological needs of college students [28], thereby reducing the frequency of smartphone use. Moreover, physical exercise can enhance dopamine concentration and the receptor binding rate in the human body and mitigate an individual’s addictive behaviors [41,42]. Globally, many studies have pointed out that smartphone overuse is closely related to people’s lack of physical exercise [43]. There is a significant negative correlation between physical exercise and mobile phone addiction [44,45,46]. Previous studies found that physical exercise has a significant negative impact on mobile phone addiction [47,48]. In view of this, this study proposes the following hypothesis:

**Hypothesis** **1** **(H1).** *Physical exercise has a significant negative effect on mobile phone addiction in Chinese college students.*

### 2.2. Psychological Resilience as a Mediator

Psychological resilience has been defined as “an individual’s ability to withstand high levels of disruptive change while exhibiting as few undesirable behaviors as possible” [49]. Early studies believe that the development of psychological resilience requires an individual to experience severe dangers in the individual’s development process, such as traumatic events or natural disasters, and excellent recovery from adversity [50]. With the deepening of research, some scholars have proposed that personal abilities such as physique, intelligence, and social skills, as well as protective factors such as interpersonal interactions and self-efficacy, also significantly affect an individual’s psychological resilience [51,52]. These abilities can be enhanced by the individual’s participation in physical exercise.

According to the challenge model proposed by Garmezy et al., moderate, controllable risks have a more positive impact on human psychological development than no risk or severe, uncontrollable risks [53]. The recreational, competitive, and open characteristics of physical exercise provide a suitable space for developing an individual’s psychological resilience [54]. Individuals will face the challenge of learning new skills, competition during physical exercise [55]. Still, as individuals experience the psychological changes of falling down and climbing up in the process of overcoming challenges, they gain more psychological resilience [29]. In physiological and psychological research, physical activity can activate the hippocampus and inactivates the prefrontal cortex, reducing the individual’s cortisol response to emotional events, thus enhancing the individual’s psychological resilience through the hypothalamic–pituitary–adrenal axis mechanism [56]. Previous studies have confirmed that physical exercise has a significant positive effect on psychological resilience [29,57]. Hence, this study proposes the following:

**Hypothesis** **2a** **(H2a).** *Physical exercise has a significant positive effect on psychological resilience in Chinese college students.*

According to the psychological resilience framework theory proposed by Kumpfe and Blath [58], psychological resilience is an essential protective factor for individuals facing problematic behaviors. People with solid psychological resilience are more capable of self-regulation, which can play a significant role in preventing problematic behaviors [59].

Studies suggest that FOMO (fear of missing out) is the primary trigger of college students’ mobile phone addiction [4,60,61]. Individuals experiencing FOMO worry about missing out on the **“**trend**”** [62] and thus engage in social surveillance, checking social media for status, photos, and video updates anytime, anywhere [63], and thus becoming dependent on mobile phones. This study proposes that college students with solid psychological resilience will self-regulate in the face of FOMO, which reduces the negative impact on themselves and thus avoids the development of mobile phone addiction. Numerous studies have shown that psychological resilience significantly reduces adverse behaviors such as gambling addiction, drug abuse, and smoking addiction [64,65,66]. In the case of mobile phone addiction, in particular, previous research shows that psychological resilience has a significant negative impact on it [59]. However, few studies have focused on psychological resilience’s role in physical exercise and mobile phone addiction. Thus, this paper proposes the following hypotheses:

**Hypothesis** **2b** **(H2b).** *Psychological resilience has a significant negative impact on mobile phone addiction in Chinese college students.*

**Hypothesis** **2c** **(H2c).** *Psychological resilience mediates between physical exercise and mobile phone addiction.*

### 2.3. Perceived Stress as a Mediator

Perceived stress has been defined as “the feelings or thoughts that an individual has about how much stress they are under at a given point in time or over a given time period, often stemming from the uncontrollability and unpredictability of one’s life” [67]. College students are vulnerable to stress in many aspects of their studies and life. High levels of perceived stress can lead to negative emotions such as anxiety and depression, which are especially common among college students [68]. The transactional theory of stress and coping believes that stress results from the interaction between the individual and the environment. Stress occurs when an individual is faced with an environment of great significance and feels an inadequacy in resources or one’s skills to cope with the environment’s needs [69,70].

For most young people, entering university is a critical turning point in life and is of great significance [71]. Simultaneously, college students are in the socio-demographic age that is prone to stress disorders [72]. They tend to regard unfavorable environmental factors such as financial difficulties, academic pressure, and interpersonal barriers in life as threats and have doubts about their abilities, leading to stress [73]. Physical exercise has been shown to enhance self-esteem and improve self-efficacy in individuals and thus is regarded as an effective way to reduce perceived stress [74,75,76]. Studies have shown a significant negative correlation between physical activity and perceived stress; people who engage in regular physical exercise experience significantly lower perceived stress [77,78,79]. Furthermore, biological studies have also shown that physical exercise can help mitigate an individual’s perceived stress [80]. Therefore, this study proposes the following hypothesis:

**Hypothesis** **3a** **(H3a).** *Physical exercise has a significant negative effect on perceived stress in Chinese college students.*

The general strain theory is extensively applied in research to analyze addictive behaviors [22]. It argues that problematic behaviors primarily result from negative emotions caused by a series of stressors (failure to achieve positively valued goals, failure to maintain positively valued stimuli, and exposure to negatively valued stimuli) [81]. College students are easily exposed to various stressors in their studies, life, and social interactions, resulting in anxiety, depression, and other emotions. As an excellent tool for passing the time and eliminating negative emotions, the smartphone [82] provides an effective way for college students to alleviate their anxiety and release stress [82,83]. Liu et al. found in a study of 899 adolescents in China that perceived stress has a significant positive impact on mobile phone addiction; the adolescents who perceived more stress were more likely to develop mobile phone addiction [22]. In addition, the COVID-19 pandemic has exacerbated the perceived stress in various groups [84]. College students perceive more significant stress from the normalized lockdowns, which can potentially increase the likelihood of mobile phone addiction. Although previous studies have confirmed that perceived stress is closely related to mobile phone addiction, few studies have focused on the mediating role of perceived stress between physical exercise and mobile phone addiction. Consequently, this study proposes the following hypotheses:

**Hypothesis** **3b** **(H3b).** *Perceived stress has a significant positive effect on mobile phone addiction in Chinese college students.*

**Hypothesis** **3c** **(H3c).** *Perceived stress mediates between physical exercise and mobile phone addiction.*

### 2.4. Psychological Resilience and Perceived Stress

According to the cognitive readiness model of psychological resilience, when individuals face daily or long-term stressful events, psychological resilience can mobilize individual resources such as positive thinking and optimism, as well as external resources such as family and peer support, prompting the individual to accept and face current difficulties and challenges and make positive cognitive and behavioral responses, thereby protecting the individual against any harm from potential negative stressors [85]. Compared with people with low psychological resilience, people with high psychological resilience possess more positive emotions and greater flexibility even in stressful situations [86,87]. The higher the psychological resilience, the better the individual’s ability and confidence to cope with stressful events, the less likely they are to be overwhelmed by stressors, and the less they perceive stress [88]. Previous studies have shown that psychological resilience has a negative predictive effect on perceived stress, and the stronger the psychological resilience, the lower the perceived stress level [88,89,90,91]. On this basis, this paper proposes the following hypothesis:

**Hypothesis** **4** **(H4).** *Psychological resilience has a significant negative effect on perceived stress.*

**Figure 1:**
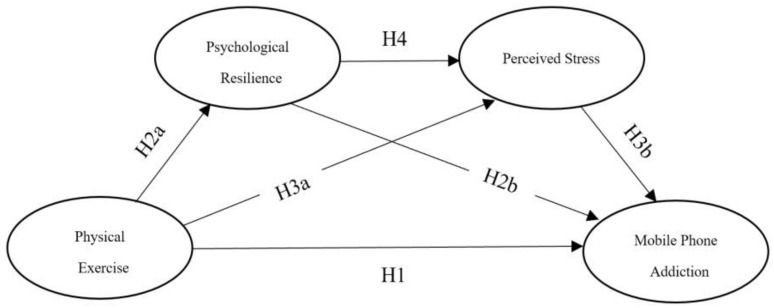
Research model.

Previous studies have not fully explored the mechanism of action between physical exercise and mobile phone addiction. This study constructed a structural equation model based on the uses and gratifications theory, the psychological resilience framework theory, and the general strain theory. It proposes that regular physical exercise can strengthen psychological resilience and reduce perceived stress in college students, thereby mitigating their mobile phone addiction (the research model is shown in Figure 1). Thus, this paper proposes the following hypothesis:

**Hypothesis** **5** **(H5).** *Psychological resilience and perceived stress play a chain mediation role between physical exercise and mobile phone addiction in Chinese college students.*

## 3. Research Methods

### 3.1. Data Collection

This study was carried out during the COVID-19 pandemic outbreak, using questionnaire surveys to collect data on college students through the Internet. We distributed 305 questionnaires at a university in Beijing and received 257 valid responses (excluding 48 questionnaires answered indiscriminately in a hasty fashion), with an effective rate of 84.3%. The basic information of the surveyed samples is shown in Table 1, which includes 73 males (28.4%) and 184 females (71.6%); the main age distribution was between 18 and 25 years (213 in total, accounting for 82.9%); the majority of the respondents were primarily undergraduates (195 in total, accounting for 75.9%).

### 3.2. Measurements

All latent variables in the questionnaire were measured using mature scales and were all self-report instruments. Simultaneously, the original English language scales were redesigned through two-way translation combined with the Chinese context to generate questionnaires that are easy for Chinese college students to comprehend.

#### 3.2.1. Physical Exercise

Physical exercise was measured using the PARS-3 scale used by Wang et al. [92]. The scale evaluates college students’ physical exercise level from three aspects: exercise intensity, duration, and frequency. The respondents were asked to measure their physical exercise on a scale from 1 to 5 on a 5-point Likert scale. The subjects’ physical activity level was calculated using the formula: the amount of physical exercise = exercise intensity × (exercise time − 1) × exercise frequency. The higher the score, the greater the individual’s amount of physical exercise. This study adopted Cronbach’s α = 0.691 (mean = 8.82; SD = ±11.43) for the physical activity scale. The total physical exercise scores of the respondents primarily concentrated in the range of 0–30, covering 240 respondents, accounting for 93.4% of the total (56 of the respondents scored 0, accounting for 21.8%); only 17 respondents scored above 30, accounting for less than 6.6% of the total.

#### 3.2.2. Psychological Resilience

This study measured college students’ psychological resilience on the Connor and Davidson Resilience Scale [88,93,94]. The scale consists of 10 items, reflecting an individual’s ability to adapt to changes, personal issues, stress, failure, etc. The 5-point Likert scale (1 point = never; 5 points = almost always) was adopted. A higher score indicates stronger psychological resilience in the individual. This study adopted Cronbach’s α = 0.91 (Mean = 3.51; SD = ±0.68) for the psychological residence scale.

#### 3.2.3. Perceived Stress

The academic community currently measures perceived stress primarily on the PSS-10 scale compiled by Cohen et al., which contains ten items [95]. This study adopted the abbreviated version of the PSS-10 scale for convenience purposes. It is composed of four items and has shown excellent reliability in previous studies [79]. Respondents were asked to recall stress they felt in the past week and rate it on the perceived stress scale, e.g., “In the past week, how many times have you felt that you have no control over the important things in your life?” The second and third question was based on reverse scoring. The 5-point Likert scale (1 point = never; 5 points = almost always) was adopted, with higher scores indicating higher levels of perceived stress. This study adopted Cronbach’s α = 0.854 (mean = 2.82; SD = ±0.89) for the perceived stress scale.

#### 3.2.4. Mobile Phone Addiction

This study measured mobile phone addiction on the MPA-11 scale compiled by Hong et al. [96]. The scale consists of 11 items and evaluates an individual’s mobile phone addiction from three aspects: (1) Time management problems (e.g., “I always wish I could spend more time using my phone”); (2) Academic problems in school and its influence (e.g., “Spending too much time on my phone has impacted my studies”); and (3) Reality substitute (“I always check my phone for missed calls or text messages before doing anything”). The 5-point Likert scale (1 point = never; 5 points = almost always) was adopted; the higher the score, the more serious the individual’s mobile phone addiction. This study adopted Cronbach’s α = 0.917 (Mean = 3.18; SD = ±0.88) for the mobile phone addiction scale.

### 3.3. Reliability and Validity Tests

This study tested the scales for reliability, validity, and common method bias with SPSS and AMOS23.0. The overall Cronbach’s α coefficient of the scales is 0.806, and the Cronbach’s α coefficient of each scale ranges from 0.691 to 0.91, all of which meet the standard requirements [97], indicating that the scales used in this study have excellent reliability (as shown in Table 2).

Confirmatory factor analysis was applied in testing the construct, convergent, and discriminant validity. In addition, in referencing the research of Wang et al. [92], the total score of the physical exercise scale was calculated and processed without a validity test. Construct validity index (CMIN/DF = 1.961; GFI = 0.845; AGFI = 0.815; IFI = 0.923; TLI = 0.914; CFI = 0.922; RMR = 0.064; RMSEA = 0.061; NFI = 0.854). The construct validity of the scales in this study is acceptable according to the recommendations [98,99]. The convergent validity is shown in Table 2. The combined reliability of the variables (CR > 0.80) and the average variance extracted (AVE > 0.50) indicate good convergent validity [100]. The discriminant validity is shown in Table 3. The correlation coefficient between variables is less than the square root of the variable’s average variance extracted, indicating excellent discriminant validity between the variables [100,101].

Furthermore, this study tested common method bias with Harman’s univariate method. Confirmatory factor analysis was performed to assess whether a single-factor latent variable model can explain all the variables of the base model [102]. Suppose the fit index of the single-factor confirmatory factor analysis (CFA) model does not meet the cutoff criteria. In that case, it suggests that there is no significant common method bias [103]. By constructing a single-factor CFA model, the fitting indices (CMIN/DF = 6.92; GFI = 0.46; AGFI = 0.37; IFI = 0.52; TLI = 0.47; CFI = 0.52; RMR = 0.16; RMSEA = 0.15; NFI = 0.48) are much lower than those of the original model. Hence, the measuring model in this study does not have a significant common method bias, meaning the verification step can proceed.

## 4. Results

This study performed the analyses using PROCESS 3.4, the macro plug-in of SPSS compiled by Hayes [104], and model 6 was selected. As shown in Table 4, physical exercise has a significant positive effect on psychological resilience (*β* = 0.195, *p* < 0.05), however its effects on perceived stress (*β* = −0.0691, *p* = 0.27) and mobile phone addiction (*β* = −0.0772, *p* = 0.17) are not significant. Psychological resilience has a significant negative effect on both perceived stress (*β* = −0.2152, *p* < 0.05) and mobile phone addiction (*β* = −0.14, *p* < 0.05). Perceived stress has a significant positive effect on mobile phone addiction (*β* = 0.4069, *p* < 0.001). Thus, hypotheses H2a, H2b, H3b, and H4 in this study have been confirmed, while hypotheses H1 and H3a have not been confirmed.

The data was bootstrap resampled 5000 times (confidence interval = 90%) to test the chain mediation effect between physical exercise and mobile phone addiction. The results are shown in Table 5. The total effect of physical exercise on mobile phone addiction was (*β* = −0.115, SE = 0.0047, *t* = −2.42, *p* < 0.05, 90%CI [−0.0193, −0.0036]), and there were three mediating pathways in the model (total mediating effect: *β* = −0.0725, SE = 0.0301, 90%CI [−0.0099, −0.0019], accounting for 63% of the total effect): (1) Physical exercise → psychological resilience → mobile phone addiction (*β* = −0.0273, 90 %CI [−0.0566, −0.003], accounting for 23.7% of the total effect). (2) Physical exercise → perceived stress → mobile phone addiction (*β* = −0.0281, 90% CI [−0.0655, 0.0066], accounting for 24.4% of the total effect). (3) Physical exercise → psychological resilience → perceived stress → mobile phone addiction (*β* = −0.0171, 90%CI [−0.0308, −0.0054], accounting for 14.9% of the total effect). The results show that psychological resilience mediates between physical exercise and mobile phone addiction, and the mediating effect of perceived stress between physical exercise and mobile phone addiction is insignificant. Still, psychological resilience and perceived stress have a significant chain mediation effect. Thus, hypotheses H2c and H5 in this study have been confirmed, while hypothesis H3c has not been confirmed.

## 5. Conclusions

### 5.1. Theoretical Implications

(1) Perceived stress and mobile phone addiction

The study results show that perceived stress has a positive and significant effect on mobile phone addiction. The stronger the perceived stress, the more severe the mobile phone addiction, which is consistent with the previous research [22,23]. The action sequence theory suggests that humans are inherently “doers” and respond instinctively to environmental stimuli [105]. When people perceive external stimuli, they will evaluate the stimuli and generate emotional responses, which triggers the need for action and drives people to make corresponding behavioral responses [106]. College students are exposed to various stressors in their studies and life. Stress stimulation usually leads to negative emotions. According to the general strain theory, individuals under stressful conditions often adopt corresponding adaptive ways to vent their negative emotions. Ames and Roitzsch [107] pointed out that perceived stress can significantly impact the individual’s craving for material possessions, i.e., individuals treat material use as an effective way to release stress. The convenience, easy access, and entertainment features that come with a smartphone can become a psychological “sanctuary” for college students, satisfying their immediate psychological need to relax and vent in the face of stress so that they can temporarily get out of the stressful situation. Over time, college students’ dependence on smartphones will evolve into mobile phone addiction.

(2) The mediating effect of psychological resilience

The study results show that physical exercise does not directly affect mobile phone addiction and that psychological resilience fully mediates between physical exercise and mobile phone addiction. First, physical exercise can positively and significantly affect psychological resilience. The higher the level of physical exercise, the stronger the psychological resilience, which is consistent with the research of Yoshikawa et al. and Ho et al. [29,57]. Second, consistent with the findings of Shen [59], psychological resilience has a significant negative impact on mobile phone addiction. The stronger the psychological resilience, the less severe the mobile phone addiction, which again confirms the essential role of the psychological resilience framework theory in preventing adverse behaviors.

The recreational and competitive characteristics of physical exercise provide a suitable space for developing psychological resilience in college students. Regular physical exercise can enhance college students’ physical fitness and interpersonal skills. Various controllable risks encountered in physical exercise can be transformed into protective factors to cultivate perseverance, improve college students’ ability to adapt to diverse environments, and help them cope with various challenges with an optimistic attitude, thereby building stronger psychological resilience. College students with strong psychological resilience are more capable of self-regulation and can effectively self-regulate in the face of mobile phone addiction-inducing factors such as FOMO. They will not follow social media excessively or fear missing out on the “trend.” Their dependence on mobile phones will be reduced, thereby avoiding the development of a mobile phone addiction.

(3) The chain mediation effects of psychological resilience and perceived stress

Consistent with previous studies [88,89,90,91], this study confirmed that psychological resilience has a negative and significant effect on perceived stress in college students; the stronger the psychological resilience, the lower the perceived stress. However, physical exercise does not directly impact perceived stress and mobile phone addiction; rather, it indirectly affects them through the mediating variable of psychological resilience. This finding is inconsistent with the studies of Yang et al. [47], Li et al. [48], and Liu et al. [22]. That is, psychological resilience and perceived stress play a chain mediating role between physical exercise and mobile phone addiction. Regular physical exercise can improve the psychological resilience of college students, thereby reducing perceived stress and mitigating mobile phone addiction.

Combining the uses and gratifications theory, this study conjectures that although both physical exercise and smartphones are effective means to satisfy people’s needs for passing the time, relaxing, and entertainment, research shows that lack of time, remote locations of exercise venues, one’s own laziness, and other unfavorable factors constitute barriers to physical exercise among college students [108]. Smartphones, nonetheless, do not come with such limitations. College students can use mobile phones for social interactions and entertainment anytime, anywhere, even when lying in bed, which satisfies their psychological needs more simply and quickly. This may be one of the reasons why physical exercise cannot directly affect mobile phone addiction.

In addition, combined with the chain mediation effect of psychological resilience and perceived stress, this study makes a second conjecture about why the direct effect of physical exercise is insignificant while explaining the chain mediation effect. Although the controllable risks of physical exercise provide a suitable space for developing psychological resilience in individuals [54], it does not follow that every college student is capable of “falling down and getting up again” when facing the challenge of learning new skills, competitions, and the frustrations of losing a game. Materialist dialectics holds that quantitative change leads to qualitative change. At the same time, the qualitative change is triggered only when the quantitative change develops to a certain extent, changing the movement of the principal contradiction within things. College students will not achieve psychological resilience when facing inadequate challenges in physical exercise or no challenge. However, with excessive challenges or the frustration of losing a game, they may develop fear and evasive behavior and remain unable to build psychological resilience. College students who face various challenges and successfully overcome them through physical exercise can achieve stronger psychological resilience, effectively self-regulate the adverse effects of various stressors, and thus avoid mobile phone addiction. In contrast, those who do not go through this process cannot develop the psychological resilience to further affect perceived stress and mobile phone addiction. Consequently, psychological resilience plays a vital role in the chain mediation model, and the indirect effect of physical exercise on perceived stress and mobile phone addiction needs to be realized through psychological resilience. This finding has contributed to the development of psychological resilience theory and research on mobile phone addiction.

### 5.2. Practical Implications

Based on the above analysis and discussion, this study puts forward the following recommendations:

(1) There are multiple ways to encourage college students to engage in physical exercise. The research results show that college students’ current physical exercise participation is relatively low. Government departments, the news media, and college administrators should publicize health and physical exercise knowledge to reduce the adverse effects of “laziness,” “lack of perseverance,” and other subjective factors on college students’ participation in physical exercise and guide them in establishing positive attitudes toward it. Simultaneously, the sports venues and facilities should be improved, college students’ sports associations should be encouraged, and a series of competitive sports should be organized on campus to provide sufficient opportunities for college students to participate in physical exercise.
(2) Giving play to the “bridge” role of psychological resilience. College administrators should focus on fostering college students’ psychological resilience through physical exercise. Sports activities with appropriate difficulty should be organized to cater to the level of student physical activity. For example, college students with low physical exercise levels can engage in sports with lower thresholds, such as frisbee. At the same time, we should pay attention to the emotional changes in college students after participating in physical exercise and encourage those who suffer frustration from an excessive challenge or competition loss to keep them from developing a fear of difficulties and evasive behavior. In this way, psychological resilience can play the “bridge role” and help college students mitigate the negative impact of stress in their studies and mobile phone addiction through physical exercise.

### 5.3. Limitations and Future Research

(1) In terms of the measuring tools, the PARS-3 scale used in this study only measures the level of physical exercise from three dimensions: duration, frequency, and intensity, which lacks comprehensiveness and accuracy. Follow-up research may reference [109] and adopt the IPAQ-SF scale to further evaluate the physical activities with different contents. Additionally, some of the items in the MPA-11 scale used in this study, such as “Before having to do something, I always check the mobile phone to see whether there are missed calls or text messages,” displayed relatively low normalized factor loadings. The reason may be that mobile phones have become an essential tool for college students in their studies and work, and checking information frequently does not necessarily constitute mobile phone addiction. Subsequent research may compile scales more compatible with the current situation in college students using the MPAI-17 scale [110] or through exploratory factor analysis.
(2) As for the research subjects, this study only focused on researching the impact of physical exercise on mobile phone addiction in college students, which is also the current primary focus in the academic community. Future research can further explore the chain mediation model of psychological resilience and perceived stress in government officials, corporate employees, etc. In addition, considering the possible impact of gender differences, future studies can be analyzed based on males and females separately. Meanwhile, adolescent females are rarely considered in the sample. Indeed, their psychological resilience and perceived stress may be different [111]. Therefore, the research about the psychological resilience and perceived stress of adolescent females can be further explored.
(3) From a research perspective, although this study revealed the role of psychological resilience as a “bridge” between physical exercise and perceived stress and mobile phone addiction, the pathway through which a critical leap from physical exercise to psychological resilience takes place, and under what circumstances physical exercise has a more significant effect on psychological resilience, remain to be explored. Hence, follow-up research can focus on the moderating effects of variables such as social support and self-efficacy in physical exercise and psychological resilience, create a moderating chain mediation model, and further explore the mechanism of physical exercise’s impact on mobile phone addiction.

## Figures and Tables

**Table 1:** Demographic of Samples.

Characteristics	Category	Sample Size	Percentage
Gender	Male	73	28.4%
	Female	184	71.6%
Age	Below 18	3	1.2%
	18–21	127	49.4%
	22–25	86	33.5%
	25–28	19	7.4%
	Above 28	22	8.6%
Educational background	Primarily Undergraduates	195	75.9%
	Master and Doctor	62	24.1%
Major	Liberal art	150	58.4%
	Science	68	26.5%
	Artistic and sports	39	15.2%

**Table 2:** Analysis of reliability and convergent validity.

Variables	*β*	α	CR	AVE
***Psychological resilience***		0.91	0.91	0.503
(1) Able to adapt to change.	0.654			
(2) Can deal with whatever comes.	0.693			
(3) Tries to see humorous side of problems.	0.674			
(4) Coping with stress can strengthen me.	0.774			
(5) Tend to bounce back after illness or hardship.	0.765			
(6) Can achieve goals despite obstacles.	0.677			
(7) Can stay focused under pressure.	0.685			
(8) Not easily discouraged by failure.	0.708			
(9) Thinks of self as strong person.	0.737			
(10) Can handle unpleasant feelings.	0.715			
***Perceived stress***		0.85	0.86	0.60
(1) In the past week, how often have you felt that you were unable to control important things in your life?	0.751			
(2) In the past week, how often have you felt confident about your ability to handle your personal problems? (reversed)	0.76			
(3) In the past week, how often have you felt that things were going your way? (reversed)	0.80			
(4) In the past week, how often have you felt difficulties were piling up so high that you could not overcome them?	0.781			
***Mobile phone addiction***		0.92	0.92	0.504
(1) While using mobile phones, I would think “just give me some more minutes”.	0.62			
(2) I have tried to decrease mobile phone usage time, but have failed.	0.638			
(3) While not using the mobile phone, I still think about using the mobile phone and have visions about using the mobile phone.	0.719			
(4) Using mobile phone at night influences my sleep.	0.627			
(5) I try to hide my mobile phone usage time.	0.658			
(6) Mobile phone usage influences my school work.	0.841			
(7) I neglect school work to spend more time on mobile phone usage.	0.875			
(8) My school performance and concentration are influenced by mobile phone usage.	0.844			
(9) Before having to do something I always check the mobile phone to see whether there are missed calls or text messages.	0.609			
(10) I find myself wanting to use the mobile phone again.	0.699			
(11) When others ask me what I am doing when I use my mobile phone, I become defensive or secretive.	0.607			

Notes: *β* is standardized loaded factor; CR is composite reliability; α is the Cronbach coefficient; AVE is average variance extracted.

**Table 3:** Analysis of discriminant validity.

	Test Score (Mean ± SD)	Psychological Resilience	Perceived Stress	Mobile Phone Addiction
Psychological resilience	3.51 (±0.68)	0.71		
Perceived stress	2.82 (±0.89)	−0.27	0.77	
Mobile phone addiction	3.12 (±0.88)	−0.25	0.48	0.71

Notes: The diagonal represented the square root of each variable AVE, and the data below the diagonal represented the correlation coefficient between variables.

**Table 4:** Hypothesis test.

Paths	β		SE	*t*	LLCI	ULCI
	Unstandardized	Standardized				
PE→MPA	−0.0059	−0.0772	0.0043	−1.37	−0.131	0.0012
PE→PR	0.0116	0.195	0.0037	3.17 **	0.0056	0.0177
PE→PS	−0.0054	−0.0691	0.0048	−1.11	−0.0134	0.0026
PR→PS	−0.2805	−0.2152	0.081	−3.46 **	−0.4143	−0.1468
PR→MPA	−0.1796	−0.14	0.0739	−2.43 **	−0.3017	−0.576
PS→MPA	0.4005	0.4069	0.056	7.15 ***	0.3081	0.4929

Notes: PE = physical exercise; PR = psychological resilience; PS = perceived stress; MPA = mobile phone addiction; β = standardized coefficient; SE = standard error; LLCI = lower limit of the confidence interval; ULCI = upper limit of the confidence interval. *** *p* < 0.001; ** *p* < 0.05; * *p* < 0.1.

**Table 5:** Indirect effects of physical exercise on mobile phone addiction.

Indirect Effect	*β*	Bootstrap SE	90% CI	Percentage Accounting for Total Effect
Total Ind	−0.0725	0.0301	−0.0099 to −0.0019	63%
Ind1	−0.0273	0.0168	−0.0566 to −0.003	23.7%
Ind2	−0.0281	0.022	−0.0655 to 0.0066	24.4%
Ind3	−0.0171	−0.0171	−0.0308 to −0.0054	14.9%

Notes: *β* = standardized coefficient; SE = standard error; CI = confidence interval; Ind = indirect effect; Ind1 = physical exercise → psychological resilience → mobile phone addiction; Ind2 = physical exercise → perceived stress → mobile phone addiction; Ind3 = physical exercise → psychological resilience → perceived stress → mobile phone addiction. The Ind is statistically significant at the 90% CI when the CI does not include zero.

## Data Availability

The data presented in this study are available on request from the first author.
